# Overexpressed Nup88 stabilized through interaction with Nup62 promotes NF-κB dependent pathways in cancer

**DOI:** 10.3389/fonc.2023.1095046

**Published:** 2023-02-09

**Authors:** Usha Singh, Divya Bindra, Atul Samaiya, Ram Kumar Mishra

**Affiliations:** ^1^ Nups and Sumo Biology Group, Department of Biological Sciences, Indian Institute of Science Education and Research (IISER), Bhopal, Madhya Pradesh, India; ^2^ Department of Surgical Oncology, Bansal Hospital, Bhopal, Madhya Pradesh, India

**Keywords:** nucleoporins (NUPs), head and neck cancer, NFkB, Nup88, Nup62

## Abstract

Bidirectional nucleo-cytoplasmic transport, regulating several vital cellular processes, is mediated by the Nuclear Pore Complex (NPC) comprising the nucleoporin (Nup) proteins. Nup88, a constituent nucleoporin, is overexpressed in many cancers, and a positive correlation exists between progressive stages of cancer and Nup88 levels. While a significant link of Nup88 overexpression in head and neck cancer exists but mechanistic details of Nup88 roles in tumorigenesis are sparse. Here, we report that Nup88 and Nup62 levels are significantly elevated in head and neck cancer patient samples and cell lines. We demonstrate that the elevated levels of Nup88 or Nup62 impart proliferation and migration advantages to cells. Interestingly, Nup88-Nup62 engage in a strong interaction independent of Nup-glycosylation status and cell-cycle stages. We report that the interaction with Nup62 stabilizes Nup88 by inhibiting the proteasome-mediated degradation of overexpressed Nup88. Overexpressed Nup88 stabilized by interaction with Nup62 can interact with NF-κB (p65) and sequesters p65 partly into nucleus of unstimulated cells. NF-κB targets like Akt, c-myc, IL-6 and BIRC3 promoting proliferation and growth are induced under Nup88 overexpression conditions. In conclusion, our data indicates that simultaneous overexpression of Nup62 and Nup88 in head and neck cancer stabilizes Nup88. Stabilized Nup88 interacts and activates p65 pathway, which perhaps is the underlying mechanism in Nup88 overexpressing tumors.

## Introduction

Nucleoporins (Nups) are the constituent proteins of the megadalton assemblies called nuclear pores. Nups form biochemically distinct and stable sub-complexes and localize to the nuclear pores and mediate nucleo-cytoplasmic transport during interphase. Interestingly, these subcomplexes disassemble, and some of them localize to chromatin and regulate mitotic spindle assembly, microtubule dynamics, and chromosome segregation in mitosis (1–3).

Nup88 forms a stable subcomplex with Nup214 and constitutes the cytoplasmic face of the nuclear pores (4). Point mutations and expression level changes in nucleoporins have links with occurrences and progression of cancer (5). Particularly, Nup88 mRNA and protein levels were reported to be enhanced in human ovarian tumors (6). Further, elevated Nup88 levels were found in several cancers irrespective of their type, degree of differentiation, or site of occurrence (7). Moreover, Nup88 levels exhibit a positive correlation with progressive stages of cancer (8). CAN (Nup214), a proto-oncogene linked with myeloid leukemogenesis (9), forms a complex with Nup88 and regulates CRM1 mediated nuclear export of macromolecules (10). Nonetheless, Nup214 is not co-overexpressed in Nup88 overexpressing cancers (11). Overexpression of Nup88 induced multinucleated phenotypes, and a multipolar spindle phenotype when depleted. Interestingly, Nup214 co-expression in Nup88 overexpressing cells ameliorated above phenotypes, highlighting the balance between free levels of Nup88 and its complexation with Nup214 in cellular homeostasis (12). Moreover, overexpression of Nup88 sequestered Nup98-Rae1 away from APC/C complex triggering early degradation of PLK1 that induced aneuploidy and tumorigenesis (13). Also, the interaction of Nup88 with Vimentin affects Vimentin organization resulting in multinucleated cells and aneuploidy (14).

Nup159, the yeast ortholog of Nup214, is mono-ubiquitinated and affects the cell-cycle progression and aneuploidy (15). In yeast, Nup88 interacted with Nup62 through the helical domain (16), and mutations in Nup62 affected the mRNA export (17). Nup62 glycosylation is an important determinant of Nup88 stability (18), and the ubiquitination of Nup88 and Nup62 affects their stability (15). Nup62 overexpression is reported from the prostate, and ovarian cancers (19, 20), and ROCK1 dependent Nup62 phosphorylation induces p63 nuclear localization and cell proliferation (21). The idea that perturbation of multiple cellular processes when Nup88 is overexpressed (22) is very general and does not provide any specific insight. Moreover, limited information about the Nup88 and Nup62 expression level changes in various cancers including head and neck cancer (11, 21) impedes our understanding of the process. Since Nup88 and Nup62 form stable complexes and their expression levels show alterations in different cancers, we have probed how the expression and interactions of Nup88 and Nup62 correlate with head and neck cancers.

Here, we report that Nup88 and Nup62 mRNA and protein levels are elevated in head and neck cancer tissues. Nup88 and Nup62 engage in a conserved interaction through their respective carboxy-terminal regions and this interaction is independent of cell cycle dynamics and glycosylation status of Nup62. Nup62 co-overexpression primarily stabilizes Nup88 and prevents its ubiquitination mediated degradation. Stabilized Nup88 interacts efficiently with NF-κB and affects proliferation, inflammation, and anti-apoptosis responses downstream of NF-κB signaling to promote tumorigenic growth.

## Results

### Nup88 and Nup62 are overexpressed in head and neck cancer

**Figure 1:**
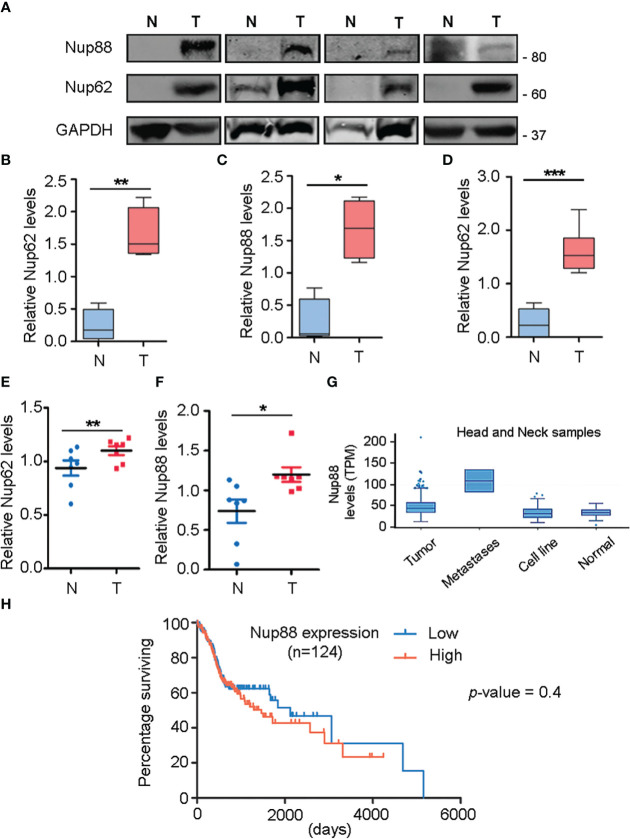
Nup88 and Nup62 are overexpressed in oral cancer. **(A)** Western blot analysis of lysates prepared from oral cancer patient tissues using antibodies against Nup88, Nup62, and GAPDH. **(B, C)** Quantification of Nup62 and Nup88 band intensities relative to GAPDH protein levels from normal and tumor tissues, shown in **(A)**. **(D)** Nup62 protein levels relative to GAPDH in oral cancer tissues (n=10) (N= Adjacent normal tissues, and T= Tumor). The asterisk represents the significance value. Values on the y-axis represent data obtained by normalizing with the GAPDH band intensity. The asterisk indicates statistical significance p<0.05. **(E, F)** Graphs indicate fold changes in Nup62 and Nup88 mRNA levels, respectively, in oral cancer tissue samples (n=7). Y-axis values indicate Nups level relative to Rps16 control levels. **(G)** Nup88 expression in TCGA head and neck statistics analyzed in Mi-Panda. **(H)** Kaplan-Meier survival curve for Nup88 using OncoLnc on oral cancer TCGA data. (Student’s t-test - paired t-test) *p < 0.05, **p < 0.01 and ***p < 0.001.

We performed a comprehensive analysis and investigated the levels of different nucleoporins in the tissue datasets available at MiPanda. The analysis revealed that Nup62 as well as Nup88 levels are upregulated in all different types of cancers analyzed, like the cancers of head and neck, breast, and stomach (**Figures  S1A, B**). We analyzed this co-upregulation of Nup88, and Nup62 in head and neck cancer tissue lysates, and observed that both Nup88 and Nup62 levels were higher in tumor tissues (**Figure 1A**, n=4). Control gene (GAPDH) levels varied among patient samples, but the ratiometric analysis of Nup62 or Nup88 with GAPDH indicates that both Nups are significantly overexpressed in oral cancer tissues (**Figures 1B, C**, n=4). Although the Nup88 and Nup62 are abundant proteins, both were poorly detected in control samples, possibly due to the limited control tissue volume. Antibodies for Nup62 and Nup88 recognize respective antigens with variable affinities. The Nup62 antibody detected antigen with much higher affinity. Accordingly, invariably higher Nup62 protein levels were detected (~2 folds) upon analysis of additional oral cancer tissues (**Figure 1D**, and S1c n=10). Using Rps16 as a loading control, we observed that Nup62 and Nup88 mRNA levels were enhanced by 1.2 and 1.6 fold, respectively (**Figures 1E, F**, n=7). Next, we analyzed the expression of Nup62 and Nup88 using the Oncomine database (23) in the Ginos Head and neck cancer statistics (24). The analysis revealed a 2.1 and 1.15 fold increase in the transcript levels of Nup62 and Nup88, respectively (**Figure S1D**). Analysis of head and neck carcinoma MiPanda database revealed that Nup88 is particularly elevated in metastatic tumors when compared to benign tumors and cell lines (**Figure 1G**). We also analyzed the co-expression of Nup62 and Nup88 in oral cancers in a collection of available datasets at MiPanda (25), and found that Nup62 and Nup88 transcript levels were significantly higher in primary tumors when compared to normal samples (**Figure S1E**). Cancer RNA-Seq Nexus (CRN) (26) analysis showed that Nup62 transcript levels are higher in progressive stages of oral cancer (**Figure S1F**). However, the Kaplan-Meier survival curve generated using Onco-Lnc (27) indicated no significant difference in survival (log rank p-value = 0.4) between low and high Nup88 expression conditions (**Figure 1H**) indicating Nup88 expression levels increasing in high grade tumors is affecting growth but no direct impact on survival.

### Overexpression of Nup88 and Nup62 can induce tumorigenic transformations

**Figure 2:**
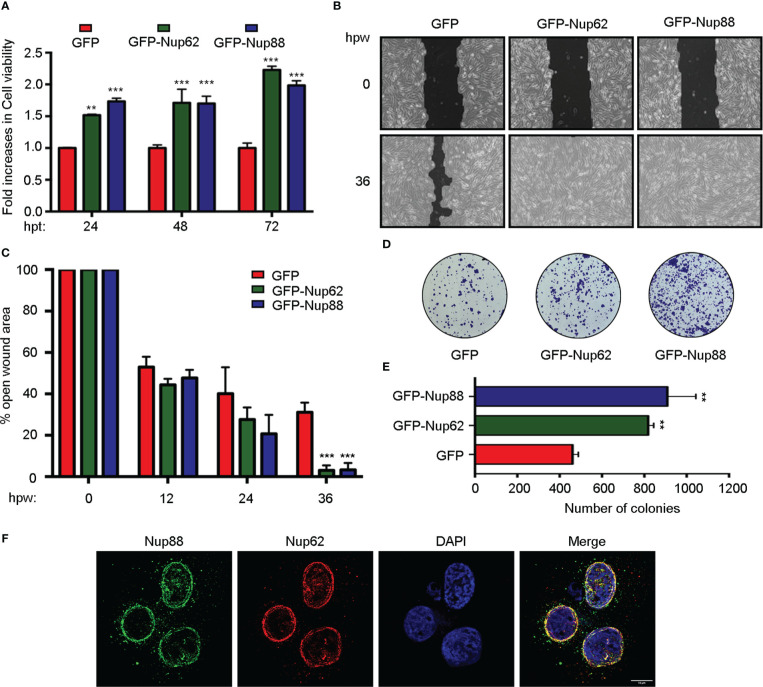
Overexpression of Nup88 and Nup62 induces tumorigenic transformations. **(A)** MTT assay based cell viability assessment in GFP, GFP-Nup62, and GFP-Nup88 transfected SCC9 cells. **(B)** Representative images of 0 h and 36 h wound healing assay in SCC9 cells expressing GFP, GFP-Nup62, and GFP-Nup88. The images were acquired at 10X magnification under an inverted microscope. **(C)** Quantification of the closure of the wound area at 0, 12, 24, and 36 h as seen in **(B)** using TScratch software. **(D)** Colony formation assay in H413 cells overexpressing GFP, GFP-Nup62, and GFP-Nup88. **(E)** Quantification of the number of colonies using Image-J/Fiji software. **(F)** Immunolocalization analysis of Nup88 and Nup62 in oral cancer cell line H413, anti-Nup88 (green), anti-Nup62 (red) and chromatin/DAPI (blue). Scale bar = 10 µm. Images are a representative from at least n=3 repeat experiments. Error bars show mean values ± SEM. Asterisks indicate statistical significance (Student’s t-test) *p < 0.05, **p < 0.01 and ***p < 0.001.

Next, we asked in a cell culture setup if Nup62 and Nup88 overexpression can contribute to vital characteristics like enhanced proliferation, migration, and loss of contact inhibition exhibited by cells in cancerous tissues. We have used SCC9 and H413 cells representing head and neck cancer for these studies. MTT assay based assessments in SCC9 cells confirmed that GFP-Nup62 and/or GFP-Nup88 expressing cells exhibit significantly increased viability (~1.5 – 2.0 folds) as compared to GFP expressing cells, suggesting an increase in metabolic activity (**Figure 2A**). The wound healing experiment was employed to assess growth and migration of cells, where a wound was created in a monolayer of SCC-9 cells and assessed for healing over different time points. Wound healing observations and its quantitation suggests that more than 95% of wound is closed by 36 hours post wounding (hpw) in GFP-Nup62 or GFP-Nup88 overexpressing cells. However, in the GFP control expressing cells, only ~60-65% wound closure was observed (**Figures 2B, C**). We assessed the loss of contact inhibition property in H413 cells expressing GFP alone or GFP-Nup62 or GFP-Nup88 using the colony-forming assay (CFA). As compared to GFP control, approximately two fold change in colony number was observed in GFP-Nup62, and GFP-Nup88 expressing cells (**Figures 2D, E**). Further, immunofluorescence analysis performed with custom generated rabbit polyclonal antibodies in H413 and SCC9 cells identified signals overlapping with Nup62 at the nuclear periphery (**Figure 2F** and **Figure S2**). Our *in cellulae* observations made with altering Nup62 and Nup88 expression levels indicated that both Nup62 and Nup88 can induce tumorigenic transformation and suggest a strong localization and interaction between Nup88 and Nup62.

### Conserved interaction between Nup88 and Nup62 occurs through their carboxy termini

**Figure 3:**
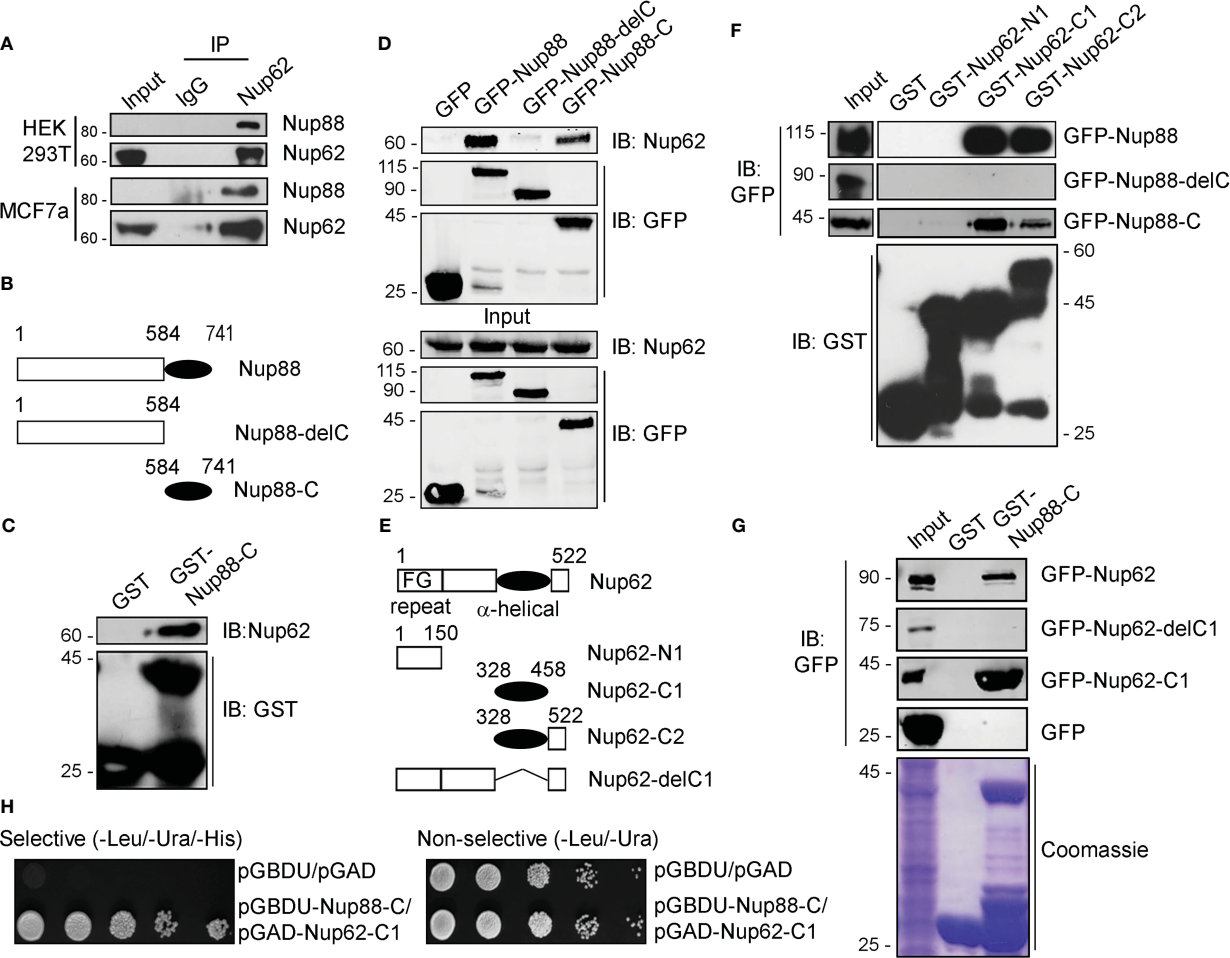
Nup88 and Nup62 interact through their carboxy-termini. **(A)** Anti-Nup62 antibody-mediated immunoprecipitation (IP) from HEK293T and MCF7 cell lysates and immunoblotting (IB) for indicated proteins. **(B)** Schematic representation of Nup88 domain constructs used in cellular transfection and GST and GFP binding protein (GBP) pulldown experiments. **(C)** Pull down of endogenous Nup62 on GST and GST-Nup88-C coated beads from HEK293T lysates and detection by indicated antibodies. **(D)** GBP pulldown from HEK293T lysates expressing either GFP-Nup88 or GFP-Nup88-delC or GFP-Nup88-C. Pulldown and input samples were probed with indicated antibodies. **(E)** Schematic representation of Nup62 constructs used in this study. **(F)** Beads coated with recombinant proteins indicated on top of the lanes used in pulldown experiments from HEK293T cell lysates expressing GFP-Nup88, or Nup88-delC or Nup88-C proteins. Pulldown samples and input fractions were immunoblotted with the indicated antibodies. **(G)** Pulldown from HEK293T cell lysate expressing GFP-Nup62, or Nup62-delC1 or Nup62-C1 or GFP on GST or GST-Nup88-C. Pulldown samples and input fractions were immunoblotted with the anti-GFP antibody. **(H)** Yeast two-hybrid interaction analysis using the DNA binding domain (pGBDU) construct of Nup88-C and activation domain (pGAD) construct of Nup62-C1. Doubly transformed yeast colonies were grown on selective and non-selective media to score for the interaction. Images are a representative from at least n=3 repeat experiments.

We asked if Nup88 and Nup62 can interact as reported in yeast (16) to mediate the Nup88 overexpression dependent cancer phenotypes. We have used HEK-293T cells to perform all relevant protein-protein interaction and other biochemical and molecular biology studies of the work reported here as HEK-293T cells are great model cell lines for expressing proteins. Similarly, the HeLa cells were used in protein localization studies by immunofluorescence due to their robust cellular components and versatile use. Accordingly, anti-Nup62 interaction antibodies mediated immunoprecipitation (IP) from HEK-293T and MCF7 cell lysates, co-immunoprecipitated endogenous Nup88 and established the robustness of this Nup88-Nup62 interaction (**Figure 3A**). For reasons beyond explanation Nup88 antibodies invariably failed to pick the Nup88 signal in input samples. Secondary structure prediction analysis on the Nup88 sequence (Uniprot ID: Q99567) suggests a much smaller coiled-coil domain mentioned in previous studies (12). To map the domains involved in Nup88-Nup62 interaction, we generated Nup88 constructs containing only the coiled-coil domain (Nup88-C) and the one lacking the coiled-coil domain (Nup88-delC) (**Figure 3B**). Using recombinant GST-Nup88-C proteins on beads, endogenous Nup62 from cell lysates was pulled down efficiently (**Figure 3C**). Similarly, GFP-Nup88 and GFP-Nup88-C immobilized on GFP-binding protein (GBP) pulled down endogenous Nup62 from HEK293T cell lysates. In a complementary observation, GFP-Nup88-delC failed to pull down Nup62 from HEK293T cell lysates (**Figure 3D**). To understand if the C-terminal alpha-helical region of Nup62 is involved in conserved interaction with Nup88, we generated Nup62 truncations (**Figure 3E**) as described elsewhere (28). GST-Nup62 truncations (N1, C1, and C2) coated GSH-beads were incubated with cell lysates expressing GFP-Nup88, GFP-Nup88-C or GFP-Nup88-delC. Both the C-terminal alpha-helical region bearing truncations, Nup62-C1 and Nup62-C2, pulled down Nup88 and Nup88-C, but the Nup88-delC could not be pulled down (**Figure 3F**). In a reciprocal pulldown, GST-Nup88-C coated beads efficiently pulled down Nup62 and Nup62-C1 but not the Nup62-delC1 (**Figure 3G**). Using the yeast two-hybrid system, we further established that the minimal coiled-coil region of Nup88 and alpha-helical region of Nup62 are sufficient to mediate the Nup88-Nup62 interaction (**Figure 3H**). Importantly, Nup62-C1 exhibited a strong and specific interaction with the Nup88 coiled-coil domain as it did not bind with a random coiled-coil domain of an intermediate filament binding protein Periplakin (PPL-C). Additionally, the Nup88-C efficiently pulled down the endogenous Nup62 and exogenously expressed GFP-Nup62-C1, but the PPL-C could not (**Figure S3**). Thus the carboxy-terminal alpha-helical region of Nup62 and the coiled-coil region of Nup88 engage in a strong Nup88-Nup62 interaction.

### Nup88 and Nup62 interaction is cell-cycle independent

**Figure 4:**
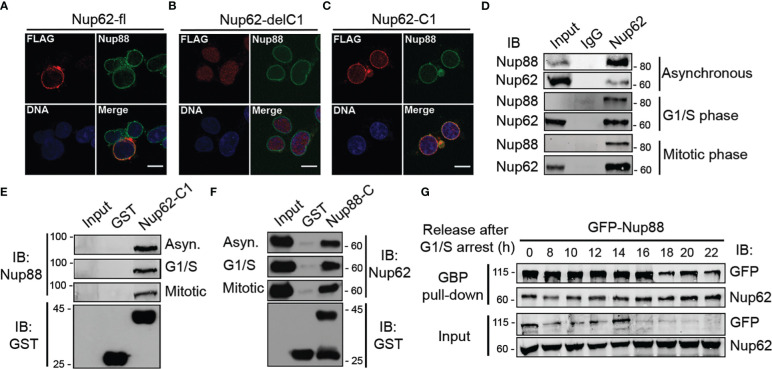
Nup88 interacts with Nup62 independent of the cell cycle phases. **(A–C)** Localization of FLAG-Nup62 constructs Nup62-fl, Nup62-delC1, and Nup62-C1 respectively in cells and detection by anti-FLAG (red) and anti-Nup88 (green) antibodies. Chromatin is stained with Hoechst 33342. Scale bar = 10 µm. **(D)** Immunoprecipitation using control and anti-Nup62 IgG from HeLa cell lysates synchronized in different phases (as indicated) of the cell-cycle. IP samples were immunoblotted with anti-Nup88 and anti-Nup62 antibodies. **(E)** Pull down on GST or GST-Nup62-C1 coated beads from HeLa lysates synchronized as indicated. Pull down material is immunoblotted with the anti-Nup88 antibody. The GST-tagged proteins were detected by anti-GST antibodies (bottom panel). **(F)** Same as in **(E)**, but the beads are coated with GST or GST-Nup88-C, and anti-Nup62 antibody used for immunoblotting. **(G)** GBP pull down from GFP-Nup88 expressing HeLa cell lysates prepared from cells released for indicated time intervals after synchronization at G1/S phase. Pull down, and input fractions were immunoblotted (IB) with anti-GFP and anti-Nup62 antibodies. Images are a representative from at least n=3 repeat experiments.

Nucleoporins exhibit cell-cycle dependent differences in their subcellular localization (29) and stability (30). The alpha-helical domain of Nup62 (Nup62-C1) assists in its centrosome localization (28). We asked if the localization and interaction between Nup88 and Nup62 changes during different cell-cycle phases. We checked the localization of Nup88 (full length, Nup88-delC, Nup88-C), and Nup62 (full length, Nup62-delC1, Nup62-C1) in cells either normally fixed (**Figure S4A**) or first extracted with Triton X-100 before fixation (pre-extraction) which allowed clear nuclear rim visualization of expressed proteins (**Figure S4B**). Full-length Nup88 and Nup62 primarily localized in the cytoplasm and at nuclear envelope, but, in a contrasting observation, Nup88-C did not localize to the nuclear envelope (NE), whereas Nup62-C1 was found at the NE (**Figure S4B**). We then asked if the NE localization of endogenous Nup88 changes under Nup62-delC1 overexpression conditions. The Nup62-fl and Nup62-C1 reactivity were strong at the nuclear rim, but the Nup62-delC1 remained diffused inside the nucleoplasm. Importantly, in all these cases, endogenous Nup88 was found in the NE (**Figures 4A–C**). We asked if the Nup88-Nup62 interaction and their protein levels exhibit any cell cycle-dependent variations. Lysates obtained from asynchronous, G1/S, and mitotic phase synchronized HeLa cells (**Figure S5**) were used in immunoprecipitation (IP) with control (IgG) and anti-Nup62 antibodies. We detected an efficient IP of Nup88 under all cell synchronization conditions (**Figure 4D**). Similarly, the GST-Nup62-C1 pulled out endogenous Nup88 (**Figure 4E**), and GST-Nup88-C pulled out endogenous Nup62 (**Figure 4F**) from asynchronous and synchronized HeLa cell lysates establishing strong and stable cell-cycle independent interaction between Nup88 and Nup62.

We next probed if the cell-cycle stages exert any effect on overexpressed Nup88. HeLa cells synchronized at G1/S boundary were released from the arrest for indicated time intervals, and GFP-Nup88 was pulled down on GBP coated beads to assess interaction with Nup62. Importantly, the interaction between Nup88 and Nup62 remained unperturbed (**Figure 4G**, first panel), indicating a strong cell-cycle stage independent interaction. Interestingly, the levels of GFP-Nup88 decreased ~18 h post G1/S release, a time-point indicative of the early G1 phase (**Figure 4G**, first and third panel). In contrast, the endogenous Nup62 levels did not change significantly (**Figure 4G**, second and fourth panels). Our data highlights the fact that Nup88-Nup62 interaction is cell-cycle independent but surprisingly overexpressed Nup88 is unstable.

### Nup62 interaction with Nup88 protects Nup88 from ubiquitination mediated degradation

**Figure 5:**
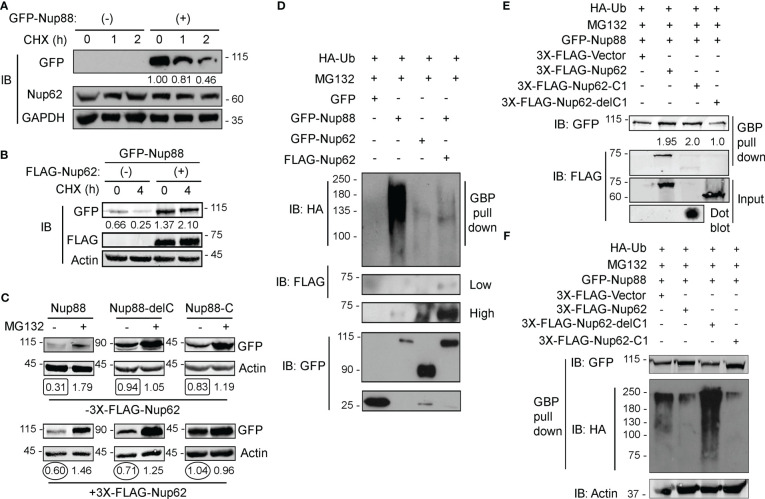
Nup62 stabilizes Nup88 protein by protecting it from degradation. **(A)** Detection of GFP-Nup88 in lysates of GFP-transfected and GFP-Nup88 transfected cells treated with cycloheximide for the indicated time points (h). Nup62 and GAPDH were used as internal loading controls and GAPDH for relative level quantification. **(B)** GFP-Nup88 transfected cells, cotransfected with 3x-FLAG-Nup62 (+), were treated with cycloheximide for the indicated time points (h). Total cell lysates were probed with anti-GFP, anti-FLAG, and anti-Actin antibodies. **(C)** HEK293T cells transfected with Nup88 constructs indicated on top of each panel was cotransfected with vector control (- 3X-FLAG-Nup62) or FLAG-Nup62 (+ 3X-FLAG-Nup62) and treated [(+) lanes], or not-treated [(-) lanes] with MG132 and lysates from these cells were immunoblotted with anti-GFP and anti-Actin antibodies. Values below each lane represent relative Nup88 levels quantified by normalizing the densitometry values of Nup88 with the respective loading control (Actin) using ImageJ. **(D)** HEK293T cells were transfected with HA-Ubiquitin and treated with MG132. These cells were cotransfected as indicated above the lanes. GBP pulldown material was immunoblotted with anti-GFP, anti-HA, and anti-FLAG antibodies. Images are a representative from at least n=3 repeat experiments. **(E)** HA-Ubiquitin transfected, and MG132 treated HEK293T cells were simultaneously cotransfected as indicated above the lanes. GBP pulldown material was probed with anti-GFP and anti-FLAG antibodies. **(F)** same as in **(E)**, and the GBP pull down material was probed with anti-HA, anti-GFP, and anti-Actin antibodies.

Reduced glycosylation of Nup62 induces Nup88 degradation (18) and we observed that overexpressed Nup88 is unstable at the onset of G1/S phase (**Figure 4G**). These observations suggest strong effects of Nup62 interaction on Nup88 stability. We, thus, probed the role of Nup62 interaction on Nup88 stability. GFP-Nup62 transfected cells (+) were treated with cycloheximide, and endogenous Nup88 levels were detected in total lysates. Although the anti-Nup88 antibody used in the study poorly detected endogenous protein, we could observe relative enrichment of Nup88 protein in GFP-Nup62 expressing cells (**Figure S6**). In GFP-Nup88 overexpressing cells, GFP-Nup88 levels decreased by ~50% (from 1.00 to 0.46) within 2 h of cycloheximide treatment, while the endogenous Nup62 levels remained unchanged (**Figure 5A**). Moreover, a distinct stabilization of GFP-Nup88 was observed (~2 folds at t= 0 h to ~8 folds at t= 4 h) when cells treated with cycloheximide also expressed FLAG-Nup62 (**Figure 5B**). These observations indicate that overexpressed Nup88 is stabilized when Nup62 is co-expressed, probably by sequestering and forming a stable complex.

We asked if Nup88 degradation is ubiquitination dependent and if the presence of Nup62 imparts stability to Nup88 against ubiquitination. Cellular levels of GFP-Nup88 and GFP-Nup88-C, capable of interacting with Nup62, increased ~ 2 folds and ~1.25 folds, respectively, when FLAG-Nup62 was coexpressed. However, the levels of Nup88-delC, unable to interact with Nup62, decreased by ~1.35 fold even when the Nup62 was co-expressed (**Figure 5C**). Subsequently, we treated HA-Ubiquitin and Nup88 and Nup62 construct expressing cells with MG132. While GFP-Nup88 expressing cells display significant ubiquitination, the GFP-Nup62 lacked any sign of ubiquitination. The Nup88 ubiquitination reversed and was undetectable when Nup62 was co-expressed (**Figure 5D**). Further, we explored the importance of Nup62 interaction on Nup88 ubiquitination. Cells co-expressing HA-Ubiquitin and GFP-Nup88 were transfected with various Nup62 constructs, and treated with MG132. GFP-Nup88 was pulled down from cell lysate on GBP beads and quantitated to assess the stability. Observations reveal that Nup62 and Nup62-C1, capable of interacting with Nup88, when expressed stabilized Nup88 (~2 fold, **Figure 5E** upper panel). In contrast, GFP-Nup88 levels were comparable to the vector and Nup62-delC1 co-expression conditions (**Figure 5E**). From a similar experimental setup (MG132 treatment of Ubiquitin and Nup88 co-expressing cells), Nup88 showed enhanced ubiquitination under Nup62-delC1 expressing conditions. However, the co-expression of Nup62 or Nup62-C1 drastically reduced Nup88 ubiquitination (**Figure 5F**). From these experiments, it is evident that Nup88 is ubiquitinated, while interaction with Nup62 reduces possibility of Nup88 ubiquitination and thus stabilizes Nup88.

### Nup88 interacts with NF-kB and affects its downstream proliferative and inflammatory pathways

**Figure 6:**
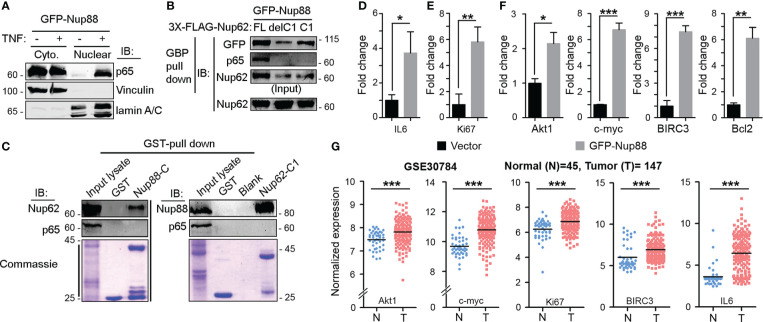
Stable Nup88 interacts with NF-κB and activates downstream pathways. **(A)** Cytosolic and nuclear fractions from GFP-Nup88 expressing cells treated (+) or not-treated (-) with TNF-α immunoblotted with anti-p65, anti-vinculin, and anti-lamin A/C antibodies. **(B)** GBP pulldown from GFP-Nup88 expressing cells cotransfected with FLAG-Nup62 constructs indicated above the lanes. The pulldown material was probed with anti-GFP, anti-p65, and anti-Nup62 antibodies. **(C)** Beads coated with GST-tagged proteins indicated on top of the wells were used in pulldown from cell lysates. Pulldown material was immunoblotted with anti-Nup62, anti-Nup88, and anti-p65 antibodies. Lower panels show Coomassie of input lysates and bead-bound samples. **(D, E)** Rps16 normalized qRT-PCR data for IL-6 and Ki67 from GFP-Nup88 transfected cells. **(F)** Actin normalized qRT-PCR data of indicated target genes from GFP-Nup88 expressing cells. All experiments were carried out in HEK293T cells. **(G)** IL-6, Ki67, Akt, c-myc, and BIRC3 expression analyzed through microarray data analysis of publically available oral cancer dataset (GSE30784) on GEO database. Error bars indicate mean values ± SEM. Asterisks indicate statistical significance (Student’s t-test) *p < 0.05, **p < 0.01 and ***p < 0.001.

Nup88-214 sub-complex and Crm1 together regulate the nuclear export of cargo proteins like NF-κB/Dorsal (p65) (31). In addition to genetic interaction, weak biochemical interaction between *Dorsal* and *mbo* (Nup88) is reported only from *Drosophila*. In HEK293T cell culture setup, we asked if Nup88 and p65 interact and whether Nup88 and Nup62 interaction has any role to play in p65 dependent functions. In this direction, first, we demonstrated that p65 redistributes inside the nucleus when GFP-Nup88 overexpressing cells are treated with tumor necrosis factor-α (TNF-α) (**Figure 6A**). Importantly, p65 was pulled down strongly on GFP-Nup88, but only when full length Nup62 was co-expressed. p65 interacted with Nup88 even when Nup62-C1 was co-expressed, but the strength of interaction was feeble. However, p65 did not interact with Nup88 under Nup62-delC1 co-expression conditions (**Figure 6B**). We further tested the involvement of interacting domains, Nup88C and Nup62-C1, in p65 pulldown. Nup88C and Nup62-C1 pulled down endogenous Nup62 and Nup88, respectively, but both failed to individually pulldown p65 (**Figure 6C**). Thus, the presence of stable Nup88-Nup62 seems imperative for interaction with p65. While analyzing the Nup88 expressing cells, we found a small fraction of overexpressed Nup88 inside the nucleus (**Figure S7A**). It is not uncommon for nucleoporins to be present in the nucleoplasm (32). Thus, we asked if nuclear Nup88 can interact and sequester p65 inside the nucleus of unstimulated cells. Indeed, the p65 was seen inside the nucleus of unstimulated GFP-Nup88 expressing cells (**Figure S7B**). We further probed if nuclear p65 is active and can induce the transcription of its target genes directly involved in the tumorigenic transformation. Comparative qRT-PCR analysis of p65 target genes in unstimulated GFP and GFP-Nup88 expressing cells suggested a significant increase in inflammatory cytokine, IL-6, levels (**Figure 6D**), and of Ki-67 (a proliferation antigen) levels (**Figure 6E**). Besides, enhancement in the expression of Akt and c-myc (growth and survival marker) and Bcl-2 and BIRC3 (apoptotic regulators) were seen (**Figure 6F**). We strengthened the observation made in cell line overexpression studies by analyzing p65 target genes in GEO & Oncomine oral cancer datasets already reported for Nup88 and Nup62 upregulation. We found the upregulation of IL6, Ki67, c-myc, Akt, and BIRC3 genes in the oral cancer dataset-GSE30784 (**Figure 6G**) as well as in the analyzed head and neck statistics available at Oncomine (**Figure S8**). Together, these observations indicate a direct interaction between Nup88 and p65, leading to the activation of the NF-κB pathway during Nup88 overexpression.

## Discussion

Nup88 overexpression is becoming synonymous with cancer progression (6). Further, it is becoming evident that levels of many nucleoporins change when in association with a disease (33). The Nup88-Nup214 complex in coordination with CRM1 regulates the transport of NF-κB and pre-ribosomal assemblies (31, 34–37). We find elevated Nup88 and Nup62 mRNA and protein levels in oral cancer tissues and a positive correlation between elevated Nup88 levels vis-a-vis poor survival rates (**Figure 1**). Possibly the co-overexpression of Nup62 and Nup88 allows the formation of a stoichiometric complex stabilizing Nup88 manifesting cancerous outcomes. While Nup88 protein levels were reported to be increasing with progressive stages of cancer (38), overexpression of no other nucleoporin could parallel these phenotypes (11). We report significantly enhanced levels of Nup88 and Nup62 protein in oral cancers. Cells overexpressing Nup88 were reported to induce multi-nuclear structures (12), and Nup62 glycosylation levels affect Nup88 stability (18). Correspondingly, elevated expression of these proteins in oral cancer cells resulted in increased cell proliferation, colony forming abilities and migration properties. These observations are in sync with reports from HeLa cells (39). While it is evident that altered expression of these nucleoporins imparts carcinogenic properties, we further need to investigate how NF-kB levels, nucleo-cytoplasmic distribution and its transcriptional factor roles are integrated when Nup88 is overexpressed.

Nucleoporins do exhibit a cell-cycle dependent difference in their stability and localization and thus are involved in cell-cycle specific interactions (34). Although Nup62 is not reported to be a stable member of the Nup88-214 subcomplex, however, *in vitro* studies with Nup62, Nup88 and Nup214 fragments (40) and the cell-cycle stage independent interaction of Nup62 with Nup88 and not perturbing the Nup88 nuclear envelope localization suggests otherwise (**Figure 4**). Similarly, the Nup107 complex exhibits cell-cycle independent interactions and mediates several functions (41). We believe that more detailed analysis needs to be performed in tissues samples but our observation from biochemical, cell biological studies suggests that overexpressed and endogenous Nup62 can form a stable subcomplex with Nup88. Interestingly, the overexpressed Nup88 when not in complex with Nup62 or Nup214 degrades over time, providing a rationale for Nup88 ubiquitination. Such stabilizing interaction is known for other proteins, including that of interaction between NF-κB and its inhibitor IκB. Thus co-overexpression of Nup62 in cancers may probably work through the stabilization of Nup88, and stable Nup88 can engage in proliferative activities inducing tumorigenic transformation.

ELYS, a dual nucleoporin, is known to affect the intranuclear dynamics of p65 (42), and *Drosophila* Nup88 (*mbo*) can perturb p65 nuclear export when the immune pathway is activated (34). While an indirect and feeble Nup88 and p65 interaction is reported in flies (34, 43), we describe a direct and strong Nup88-p65 interaction (**Figure 6**). We also suggest that Nup62 co-expression can stabilize overexpressed Nup88 and strengthens the Nup88-p65 interaction.

**Figure 7:**
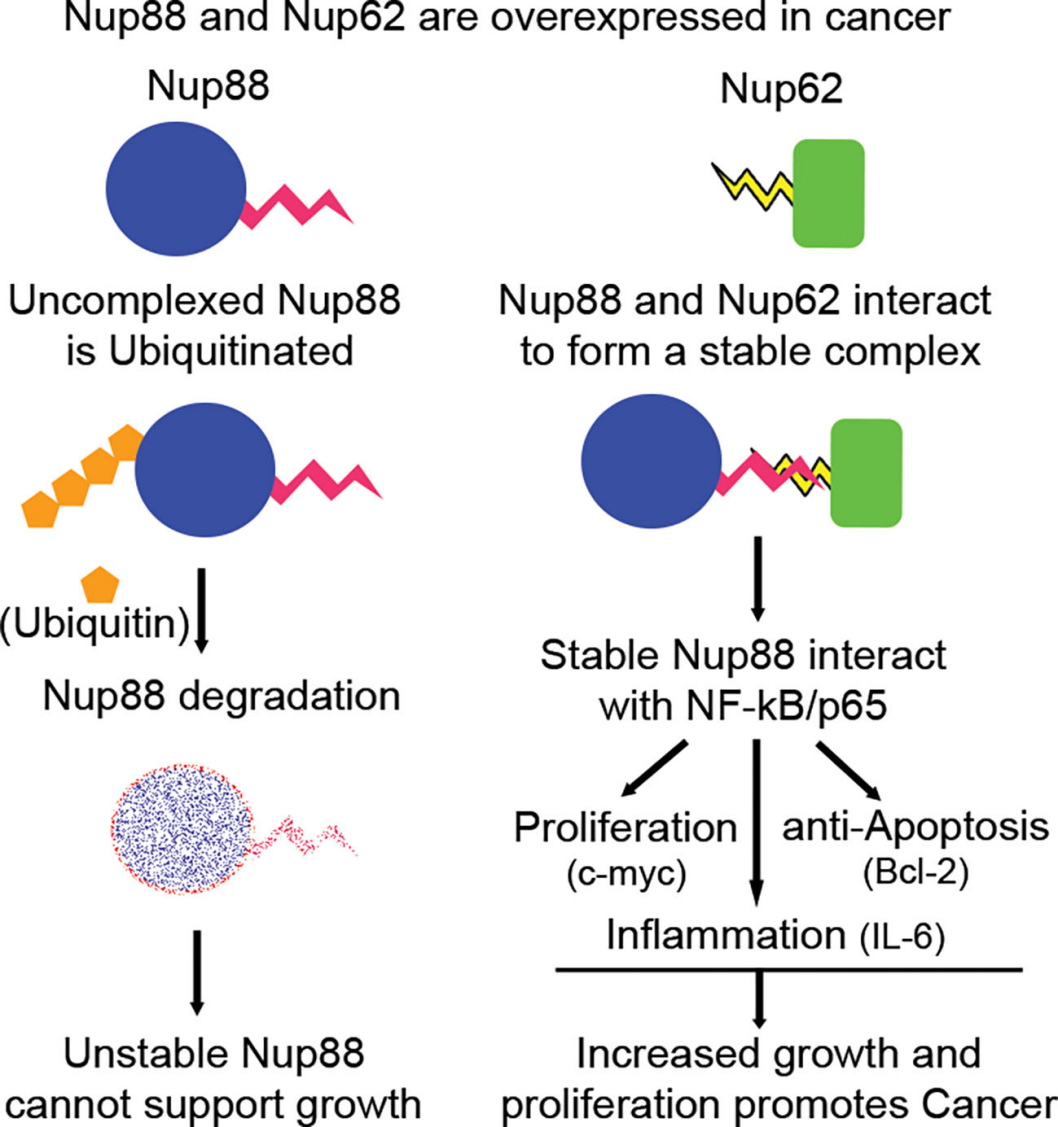
Nup88 in complex with Nup62 is stable and affects growth, proliferation and survival arm of NF-κB pathway in cancer. Nup88 and Nup62 are overexpressed in cancer, including head and neck cancer. Excess Nup88 in these cancer tissues can be targeted for degradation by ubiquitin-mediated proteasomal pathway, whereas Nup88 when complexed with co-expressed Nup62 in these tissues is stable. Stabilized Nup88 interacts with p65 and sequesters active p65 inside the nucleus. The extended presence of p65 inside the nucleus under Nup88 overexpressing conditions promotes the expression of target genes like c-myc, Bcl2, and IL-6 to simultaneously regulate growth, proliferation, apoptosis, and inflammation driving tumorigenesis.

NF-κB signaling plays an important role in regulating cell proliferation, apoptosis, and inflammation (44), moreover, the inflammatory milieu is known to support tumor growth and progression (45). Nup88-p65 interaction induces the expression of the right combination of pro-growth and anti-apoptotic molecules supporting tumorigenesis. The unique presence of NF-κB in the nucleus of unstimulated Nup88 overexpressing cells favored production of inflammatory cytokines like IL-6 and other survival signals capable of inducing neoplastic transformations (**Figure 6**). Coincidently, this observation aligns well with the IL-6 expression and inflammatory milieu in cancer (46). Our observation is in coherence with nucleoporins affecting the EGFR signaling pathway (47) and multiple roles attributed to overexpressed Nup88 inducing cancerous growth. Together, our data indicate that when cancers overexpress Nup88, often Nup62 is co-expressed. It allows Nup88 stabilization and perhaps deregulation of the NF-κB transcriptional paradigm. The dysregulated Nup88-NF-κB axis is inclined towards p65 dependent inflammatory and pro-growth axis. In agreement with this, a recent report suggests multiple roles for overexpressed Nup88 (22). We propose that NF-κB pathway activation seems to be one of the mechanisms operating in Nup88 overexpressing cancer (**Figure 7**).

## Material and methods

### Cancer tissue collection

Head and neck cancer tissue samples (T) and adjacent normal tissues (N) were collected from Bansal Hospital, Bhopal, India. The study was approved by the Institute Ethics Committee of Indian Institute of Science Education and Research (IISER) Bhopal (IEC approval document # IISERB/IEC/2016/meetings/05/04) and samples were collected with the consent of the patients. The tissues were snap-frozen immediately after surgery and stored at -80° C until use. The tissues for RNA isolation were collected in RNA Later (Thermo Fisher Scientific, # AM7024). The clinical characteristics of patients used in the study are listed in **Supplementary Table 1**.

### Bioinformatics analysis

The co-expression plot for Nup88 and Nup62 in normal, primary cancer, metastasis and cell lines for oral cancer was extracted from MiPanda (http://mipanda.org). The cancer stage-specific expression was analyzed using Cancer RNA-Seq Nexus (CRN) (http://syslab4.nchu.edu.tw/). The differential expression graph for both the genes was plotted using GraphPad Prism. The survival curves specific to Nup88 and Nup62 were obtained from OncoLnc (http://www.oncolnc.org). The differential expression pattern of Nup88 and Nup62 in oral cancer was analyzed with the help of Oncomine database. The graph of analyzed expression data of Nup88 and Nup62 in oral cancer was saved for the representation.

### Plasmids

The full-length construct of Ubiquitin (HA-Ubiquitin Plasmid # 18712) and Nup88 (pEGFP-Nup88 Plasmid # 64283) were obtained from Addgene. The C-terminal coiled-coil domain (Nup88-C, amino acids 585-741) of Nup88 was PCR amplified from human testis cDNA and cloned into pGEX6P1 with EcoRI and SalI enzyme sites. This domain was further subcloned into the pEGFP vector. The Nup88 construct lacking the coiled coil domain (Nup88-delC) was created by inserting a stop codon after 584^th^ amino acid through site directed mutagenesis (Q5 Site Directed Mutagenesis Kit, NEB-E0554S) using pEGFP-Nup88 as template. pEGFP-Nup62 was a kind gift from Dr Radha Chauhan (NCCS, Pune). The N-terminal and C-terminal truncations of Nup62 were PCR amplified and cloned into pGEX6P1, pCMV3Tag1a and pEGFP vectors. The yeast-two hybrid constructs for Nup88 and Nup62 truncations were made by subcloning them into pGADC1 and pBDUC1 vectors. Nup88-C (aa 585-741) and Nup62-C1 (aa 328-458) coding sequence were inserted into pGADC1 vector harboring GAL4 activation domain (AD) and pBDUC1 vector harboring GAL4 DNA binding domain (BD).

### Reagents and antibodies

Reagents and antibodies used in this study were purchased from miscellaneous sources. Cycloheximide (MP Biomed, # 100183) was used at 1 µg/ml for different time points. MG132 (HiMedia, 474787-10MG) was used at 10 µM concentration for 8 h. The antibodies used for western blotting are anti-Nup88 (BD Biosciences, # 611896, 1:2000 dilutions), anti-Nup62 (BD Biosciences, # 610497, 1:6000 dilutions), anti-GAPDH (Abgenex, # 10-10011, 1:6000 dilutions), anti-GFP (Santa Cruz, # 9996 1:5000 dilutions), anti-GST (1:500 dilutions), anti-HA (Sigma, # H6908, 1:2000 dilutions), anti-FLAG (Sigma, # F7425, 1:2000), anti-Actin (BD Biosciences, # 612656, 1:5000 dilutions), anti-Lamin A/C (BD Biosciences, # 612162, 1:2000 dilutions), anti-NFκB p65 (BD Biosciences, # 610868, 1:2000 dilutions), goat anti-rabbit IgG-HRP (GeNei, # 114038001A, 1:10000 dilutions), goat anti-mouse IgG-HRP (GeNei, # 114068001A, 1:10000 dilutions). A polyclonal antibody was generated against Nup88 by immunizing rabbits with Nup88 fragment (aa 1 - 584) lacking the C-terminal coiled coil-domain as an antigen. Antibodies were purified from immunized serum over NHS-Sepharose beads immobilized with appropriate antigen.

### Cell culture

HEK293T, HeLa, SCC9, and MCF7 cells were obtained from American Type Culture Collection (ATCC). The H413 cells were obtained from Sigma Aldrich. HEK293T, HeLa, and MCF7 cells were grown and maintained in Dulbecco’s Modified Eagle’s Medium (DMEM, Gibco, # 11995-065), while SCC9 and H413 cells were grown and maintained in DMEM nutrient mixture F12 (Thermo, # 11320082), supplemented with 10% fetal bovine serum (FBS, Invitrogen, # 16000044) and antibiotics (100 units/ml Penicillin and Streptomycin, Invitrogen, # 15140122) in a humidified incubator with 5% CO_2_ at 37° C.

### Cell synchronization and FACS analysis

HeLa cells were synchronized in G1/S phase by double thymidine block and into M-phase by a thymidine block followed by nocodazole treatment. Cells were cultured at 30% confluency, and two cycles of 2 mM thymidine was added for 18 h with 9 h post-release between the two treatments to synchronize cells at G1/S boundary. For the M-phase block, cells were cultured at 40% confluency and treated with 2 mM thymidine for 24 h. Cells were then released for 5 h and treated with 100 ng/ml nocodazole for 12 h. Shake-off method was used to collect the mitotic cells. For FACS analysis, HeLa cells blocked in G1/S were trypsinized, and the cells blocked in M-phase were collected by mitotic shake-off, washed twice with PBS, and fixed in 70% ethanol at -20° C for 12 h. Fixed cells were resuspended in PBS containing 50 µg/ml each of RNase A and propidium iodide. The cell cycle distribution was acquired by BD Calibur flow cytometry and analyzed by Modfit LT software.

### Lentivirus production

HEK293T cells were transfected with pLKO.1 shRNA plasmid (Sigma, Mission human genome shRNA library) and packaging plasmids- delta 8.9, VSV-G in a ratio of 10:5:1 with polyethylene-imine (PEI) following the standard protocol. After 12 h the media was replaced with fresh DMEM media containing 10% FBS and antibiotics. After 24 h and 48 h the supernatant was collected and spun to remove the cellular debris. The supernatant was filtered through a 0.45 µm filter and stored at -80° C until further use.

### RNA interference and quantitative RT-PCR

Total RNA from cultured cells or tumor tissue was extracted by TRIzol (MP Biomed, #15596018) method. The genomic DNA contamination was removed by RNAse free DNAse. 1 µg of RNA was reverse transcribed to cDNA by iScript cDNA synthesis kit (Bio-Rad, #17088) as per the manufacturer’s instructions. RT-PCR was performed using SYBR Green PCR master mix on a (BIO-RAD CFX Connect™ Real-Time System) Rps16 and Actin gene was used as the control gene, and the relative transcript level was calculated by CT value (2^-ΔΔCT^). Student’s t-test was used to compare the differences in the gene expression, and p-value < 0.05 was considered significant. The primers used are listed in **Supplementary Table 2**.

### Cell fractionation

Cells were harvested after the respective treatment and washed twice with 1X PBS and resuspended in Extraction Buffer A (10 mM HEPES, 1 mM EDTA, 1 mM EGTA, 10 mM KCl, 1 mM DTT, 5 mM NaF, 1 mM Sodium vanadate, 10 mM Sodium molybdate, 0.5 mM PMSF and 1X Protease inhibitor cocktail) and was incubated on ice for 15 min. 0.3 µl of 10% NP-40 was added to it and vortexed for 30 sec at 4° C. The lysate was centrifuged at 10000xg for 1 min at 4° C. The supernatant was collected as the cytosolic fraction. The pellet was resuspended in Extraction Buffer B (20 mM HEPES, 1 mM EDTA, 1 mM EGTA, 400 mM NaCl, 1 mM DTT, 5 mM NaF, 1 mM Sodium vanadate, 10 mM Sodium molybdate, 0.5 mM PMSF and 1X Protease inhibitor cocktail). It was incubated for 30 min on a shaker at 4° C and then centrifuged at 20,000xg for 5 min. The supernatant was collected as nuclear fractions.

### Western blot

HEK293T, MCF7, SCC9 and HeLa cells were lysed in RIPA buffer (50 mM Tris pH 7.5, 10 mM EDTA, 1 mM EGTA, 150 mM NaCl, 1% Triton X 100, 0.2% Sodium deoxycholate and 1X Protease Inhibitor (Amresco, # M250). Cell lysates were sonicated and centrifuged at maximum speed (14,800 rpm) to collect the supernatant. The homogenized head and neck tissues were lysed in GLyse AT buffer (GCC BIOTECH, # GPA-004). The total protein is quantified using Bradford assay, and samples were prepared by adding 6X SDS sample buffer and boiled for 10 min at 100° C. The electrophoresed protein samples were transferred to polyvinylidene difluoride membrane (PVDF) and blocked with 5% (w/v) non-fat milk and probed with suitable primary and secondary antibodies. The bands were detected with enhanced chemiluminescence substrate (BIO-RAD Clarity™ Western ECL Substrate, # 170-5060) method or by using the Odyssey infrared imaging system (LICOR Odyssey).

### Immunoprecipitation

The confluent HEK293T, MCF7, SCC9 and HeLa cell monolayer was lysed in 500 µl of RIPA buffer, sonicated and centrifuged at maximum speed. The collected supernatant was incubated with 5 µg of anti-Nup62 and anti-mouse IgG and incubated at 4° C on rocker 12 h. 20 µl of Protein-G sepharose beads were added to it and further incubated at 4° C on a rocker for 4 h. Bead bound samples were centrifuged, and unbound fractions were collected separately, and beads were washed four times with chilled PBS. The eluted protein samples were processed with 6X SDS sample buffer, and samples were analyzed by western blotting as described earlier.

### GST pulldown assay

The GST and GST-fused proteins were purified from bacterial strains- *E. coli* BL21DE3 Star and Codon plus cells. 20 µg of each protein was allowed to bind glutathione beads for 1h at 4° C on a rocker. The unbound protein was removed, and the beads were washed 4 times with a wash buffer (20 mM Tris, 150 mM NaCl and 1 mM EDTA). The pulldown was performed by adding 500 µg of HEK293T or HeLa cell lysate (asynchronous or synchronous, depending on the experiment) and allowed to bind for another 1 h at 4° C on a rocker. The unbound fraction was removed, and washes were given as above. The eluted protein was analyzed by western blotting.

### GBP pulldown assay

The experiment was performed as described previously (48). In brief, HEK293T cells were transfected with pEGFP-Nup88, pEGFP-Nup62, and their truncations. 48 h post-transfection cells were harvested, lysed, sonicated, and centrifuged. The supernatant fraction was incubated with GST-GBP protein at 4° C on the rocker for 12 h. 20 µl of glutathione beads were added and incubated at 4° C on a rocker for 4 h. Further washing and elution steps are similar to the GST pulldown experiment.

### Yeast two-hybrid assay

The pGADC1 and pGBDUC1 constructs of Nup88 and Nup62 truncations were co-transformed into yeast two-hybrid strain PJ69-4A. Double transformants were obtained on a non-selective (lacking Leu and Ura, double drop out) media. The ten-fold serial dilutions of equivalent numbers of transformants were spotted on non-selective (lacking Leu and Ura, double drop out) and selective media (lacking Leu, Ura, and His, triple drop out) and incubated at 30° C for three days for transformants to appear.

### Cell viability assay

SCC9 cells were transfected with pEGFP, pEGFP-Nup88, and pEGFP-Nup62 in a six-well culture plate. 24 h post-transfection cells were harvested, and 5x10^3^ cells were seeded in each well of a 96 well culture plate in triplicates and allowed to grow for another 24 h, 48 h, and 72 h. The cell growth was measured by the conversion of MTT-tetrazolium salt to formazan crystal. 20 µl of MTT (2 mg/ml) was added to each well, incubated for 4h, and the reaction was terminated by adding 100 µl of DMSO. Viability and cell proliferation were assessed by measuring the optical density at 570 nm in a plate reader (BioTek Eon, 11-120-611).

### Wound healing assay

Oral cancer cells (SCC9) were transfected with pEGFP, pEGFP-Nup88, and pEGFP-Nup62 in a six-well culture plate setup. At 90% confluence, a scratch was created with a 10 μl pipette tip. The cellular debris (dislodged cells) was removed by thorough PBS washing. The cells were imaged at 12, 24 and 36 h intervals on an inverted microscope (Leica Microsystems Model- DMIL LED Fluo). The wound closure rate in each case was measured from images using TScratch software (49).

### Colony-forming assay

H413 cells were transfected with pEGFP, pEGFP-Nup88, and pEGFP-Nup62 in a six-well culture plate. After 24 h of transfection, cells were harvested and seeded (2000 cells/well) in a six-well culture plate and were allowed to grow for 15 days. The cells were washed with PBS and imaged on an inverted microscope (Leica Microsystems Model- DMIL LED Fluo) to determine the colony size and area. Number of the colonies obtained in each case was determined with ImageJ software, and the graph was plotted using GraphPad. Further, cells were fixed in 3:1 ratio of methanol: acetic acid for 5 min. After fixation, cells were stained with 0.05% crystal violet in methanol for 15 min and washed with water to remove excess stain. The crystal-violet stained images were captured with a camera and used for the representation.

### Immunofluorescence

HeLa, H413 and SCC9 cells were grown in a six-well culture plate with coverslips. Adhered HeLa cells were washed with PBS and pre-extracted using PHEM buffer (60 mM PIPES, 20 mM HEPES, 10 mM EGTA, 0.2% Triton X-100 and 4 mM MgSO_4_) for 5 min at room temperature (RT). The H413 and SCC9 cells were washed with PBS and pre-extracted with 0.05% Digitonin for 2-5 minutes at RT. Pre-extracted cells were fixed with 4% paraformaldehyde for 15 min and permeabilized with rehydration buffer (10 mM Tris pH 7.4, 150 mM NaCl, 0.1% Triton X-100) for another 15 min at RT. The cells were blocked using 5% normal goat serum for 30 min and incubated with corresponding primary antibody (anti-Nup88, 1:200 and anti-Nup62, 1:1000) at 4° C for 12 h. After three PBS washes, 1:800 dilutions of Alexa-fluor conjugated secondary antibody (Alexa Fluor 488, Invitrogen, # A11034, Alexa Fluor 568, Invitrogen, # A11031) was added to the cells and allowed to incubate for an hour at RT. Again, three washes of PBS were given, and nuclei were stained with Hoechst 33342 (Invitrogen, # H1399, 1 mg/ml, 1:5000 dilutions). The coverslips were mounted on slides with VECTASHIELD mounting medium (# H1000), and images were captured on a Zeiss LSM 780 Confocal Microscope and Olympus Confocal Laser Scanning Microscope-FY3000. All images were analyzed using ZEN (Zeiss) or Image J/Fiji software.

### Statistical analysis

The statistical analysis was performed with GraphPad Prism5. Student’s t-test and two-way ANOVA were used to calculate the significance value. In the bar graphs, differences between two groups were compared using an unpaired two-tailed Student’s t-test or Dunnett’s multiple comparisons test. In case of cancer tissue analysis, paired two-tailed Student’s t-test was used to study the significance. The differences were considered statistically significant with *p<0.05, ** p<0.01, and *** p<0.001.

## Data availability statement

The original contributions presented in the study are included in the article/**Supplementary Material**. Further inquiries can be directed to the corresponding author.

## Ethics statement

The studies involving human participants were reviewed and approved by Institute Ethics Committee of IISER Bhopal and Ethics Committee of Bansal Hospital. Written informed consent to participate in this study was provided by the participants’ legal guardian/next of kin.

## Author contributions

US and DB designed and performed all experiments, analyzed and interpreted data. RM helped in experiment designing and data interpretation. US, DB, and RM wrote the manuscript. AS provided cancer patient’s tissue and patient data for all relevant analyses.
