# Supplementary material for: Overexpressed Nup88 stabilized through interaction with Nup62 promotes NF-κB dependent pathways in cancer
Source: Front Oncol. 2023 Feb 9;13:1095046. doi: 10.3389/fonc.2023.1095046 (PMC9947638; doi:10.3389/fonc.2023.1095046)
Supplement: Supplementary file 1 [file DataSheet_1.pdf]

Supporting information

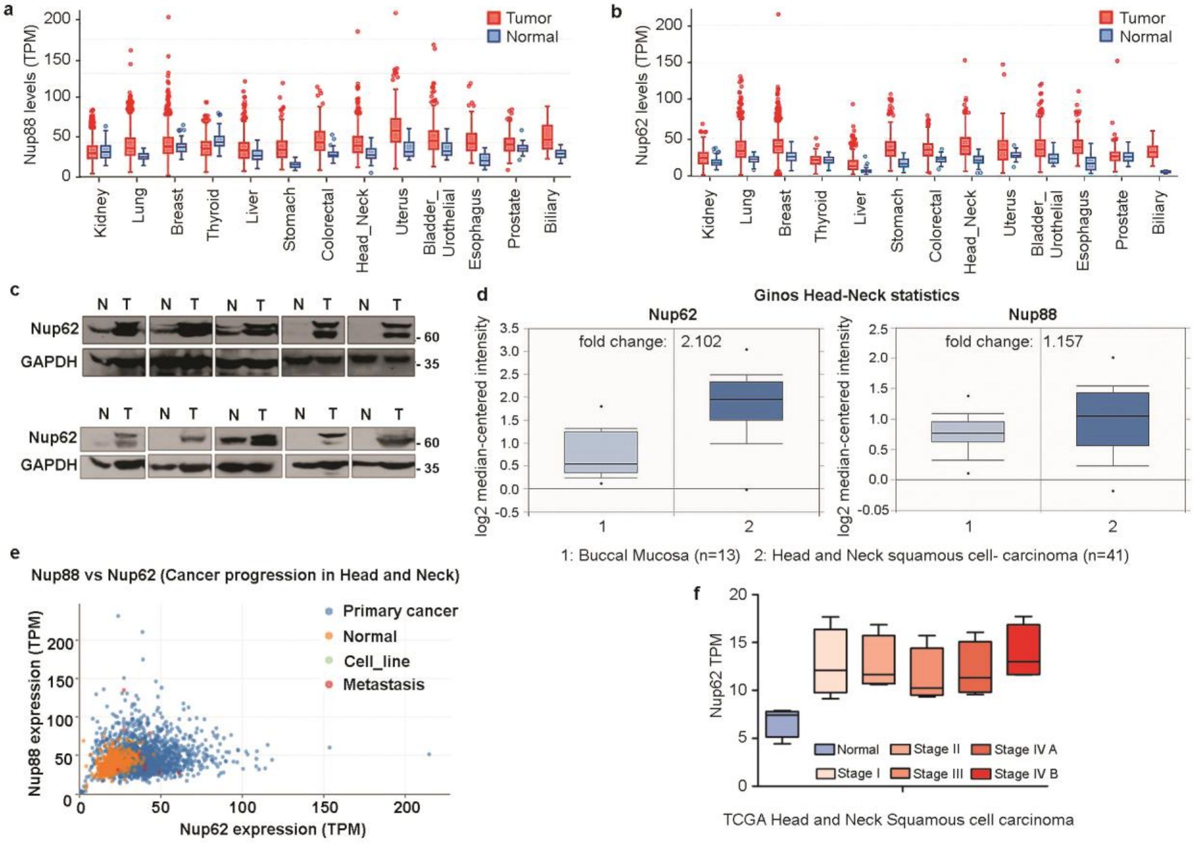

Fig. S1: Nup88 and Nup62 expression in head and neck cancer

Analysis of gene expression level variations for Nup88 (a), and Nup62 (b) as boxplots in various cancer datasets listed at MiPanda. Y-axis represents transcript level as transcript per million (TPM) (c) Western blot analysis of lysates prepared from oral cancer tissues using antibodies against Nup62, and GAPDH (N=Normal adjacent tissues, T=Tumor) (d) Nup62 and Nup88 expression analysis in head and neck cancer. Nup62 and Nup88 expression in Ginos head and neck statistics analyzed in Oncomine (*t–test*, *p<0.05*). (e) Co-expression analysis of available cancer datasets for Nup88 and Nup62. The plot is generated through Mi-Panda. (f) TCGA data for Nup62 TPM (Transcript per Million) in different head and neck cancer stages analyzed using Cancer RNA-Seq Nexus (*one-way analysis*, *p<0.05*).

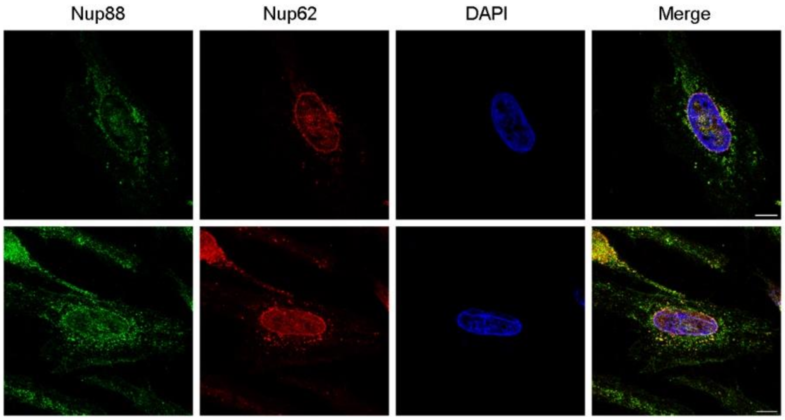

Fig. S2: Immunolocalization of Nup88 and Nup62 at nuclear envelope in SCC9 cells.

Digitonin pre-extracted SCC9 cells were probed with anti-Nup88 (1:200, rabbit polyclonal, green) and anti-Nup62 (1:1000, mouse monoclonal, red) antibodies. Chromatin is stained with DAPI (blue), and the scale bar = 10 µm.

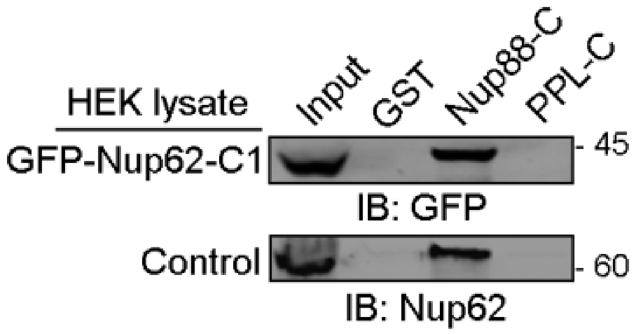

Fig. S3: Nup88 coiled-coil interaction with Nup62 is specific

Pulldown from control (untransfected) and GFP-Nup62-C1 transfected HEK293T cell lysates on GSH beads coated with recombinant GST-tagged proteins as indicated on top of each lane. The pulldown material was immunoblotted with anti-GFP and anti-Nup62 antibodies.

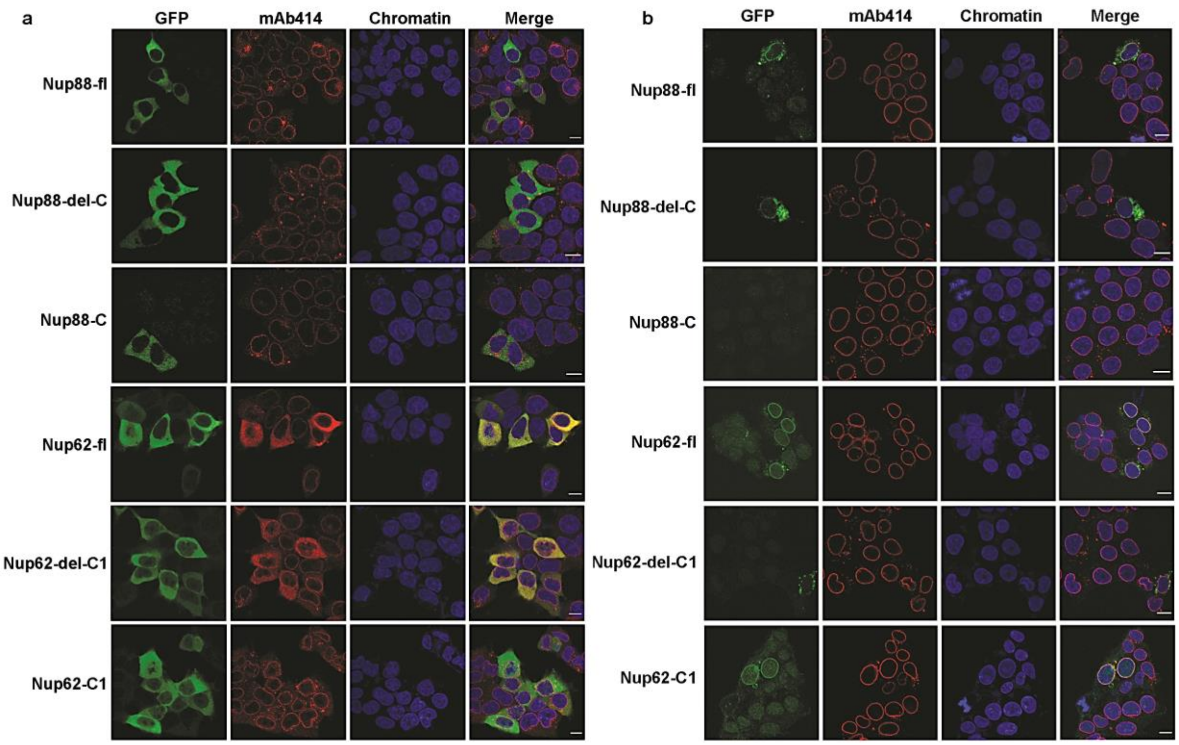

Fig. S4: Localization of Nup88 and Nup62 constructs in cells

HeLa cells transfected with the indicated GFP-tagged Nup88 constructs- (Nup88-fl, Nup88-delC, Nup88-C) or Nup62 constructs - (Nup62-fl, Nup62-delC, Nup62-C1). Cells were either not treated (non-pre-extracted), (a), or treated (pre-extracted), (b) with Triton-X-100 prior to fixation. Cells were immunostained with anti-GFP (green) and FG-Nups recognizing mAb414 (red) antibodies. DNA is stained with Hoechst 33342. Scale bar = 10 µm.

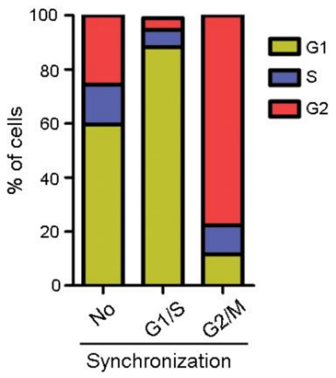

Fig. S5: Synchronization of cells

Graphical representation of FACS data under different treatments indicating the percentage of cells synchronized in different phases of the cell cycle.

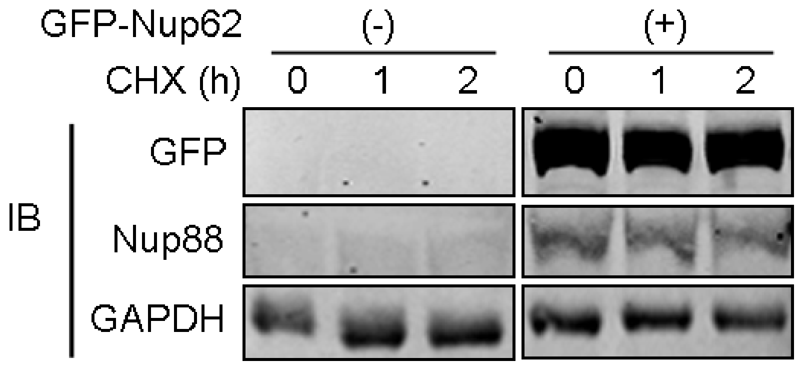

Fig. S6: Nup88 is stabilized in presence of Nup62

GFP-Nup62 expressing (+) or lacking (-) cells were treated with cycloheximide for the indicated time points. Total lysate prepared from GFP transfected cells (left panels) and GFP-Nup62 transfected cells (right panels) were immunoblotted with anti-GFP, anti-Nup88, and anti-GAPDH antibodies.

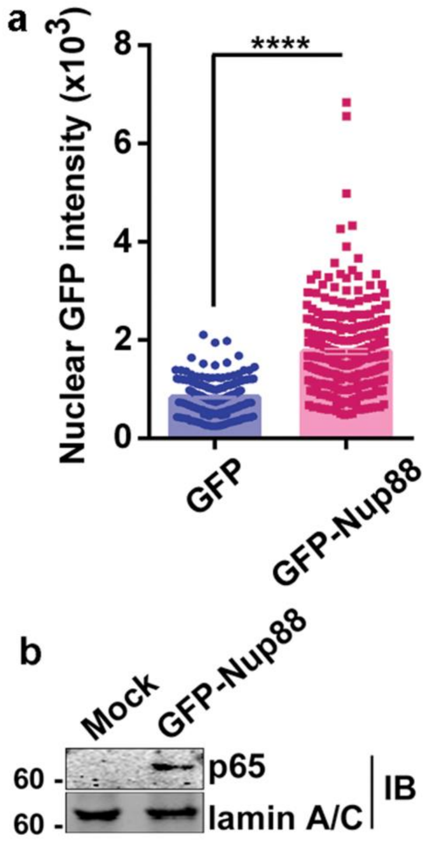

Fig. S7: Nuclear localisation of Nup88 sequesters p65 into the nucleus

(a) Quantification of nuclear GFP signal intensities from GFP and GFP-Nup88 transfected HeLa cells using ImageJ/Fiji Software. The graphs were plotted in GraphPad Prism5 and statistical significance was calculated from Student's t-test. Asterisk represents the significance value, *****p* <0.0001 (b) Nuclear fractions from control and GFP-Nup88 transfected cells immunoblotted with anti-p65 and anti-Lamin A/C.

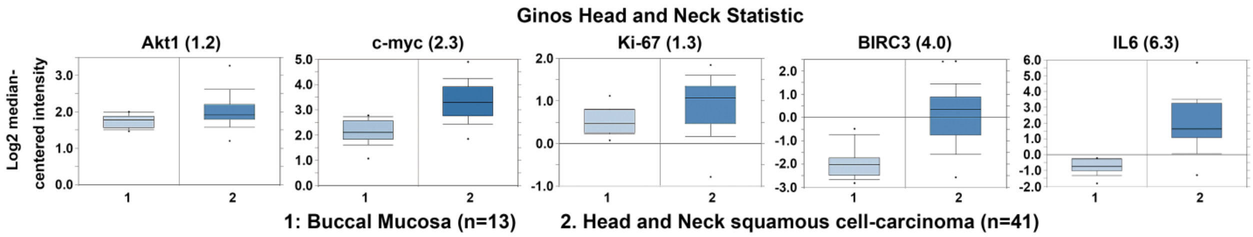

Fig. S8: p65 target genes analyzed in Ginos Head and Neck Statistics at Oncomine.

Akt, c-myc, Ki67, BIRC3, and IL-6 expression analyzed at Oncomine. The boxplots were downloaded from Oncomine for representation. The fold change for each gene is mentioned in the bracket above the plots.

Supplementary Table

The table 1 represents the clinical characteristics of oral cancer patients and table 2 enlists the primers used for qRT-PCR.

Supplementary Table 1: Clinicopathological characteristics of head and neck cancer patients.

Sample ID	Gender	Age of Patient	Site of Cancer	Histopathology
S-1	Male	49	Lower Lip	Squamous Cell Carcinoma (SCC)
S-2	Male	29	Buccal Mucosa	Squamous Cell Carcinoma (SCC)
S-3	Female	60	Lower GBS	Squamous Cell Carcinoma (SCC)
S-4	Female	46	Buccal Mucosa	Squamous Cell Carcinoma (SCC)
S-5	Male	45	Buccal Mucosa	Squamous Cell Carcinoma (SCC)
S-6	Male	55	Buccal Mucosa	Squamous Cell Carcinoma (SCC)
S-7	Male	61	Tongue	Coagulative necrosis
S-8	Female	58	Tongue	Squamous Cell Carcinoma (SCC)
S-9	Male	60	Buccal Mucosa	Squamous Cell Carcinoma (SCC)
S-10	Male	47	GBS	Squamous Cell Carcinoma (SCC)
S-11	Male	70	Buccal Mucosa	Verrucous Carcinoma
S-12	Male	53	Buccal Mucosa	Squamous Cell Carcinoma (SCC)
S-13	Male	48	GBS	Squamous Cell Carcinoma (SCC)
S-14	Male	42	Oral Cavity	Squamous Cell Carcinoma (SCC)
S-15	Male	42	Buccal Mucosa	Verrucous Carcinoma
S-16	Male	39	Tongue	Squamous Cell Carcinoma (SCC)
S-17	Female	38	Buccal Mucosa	Squamous Cell Carcinoma (SCC)
S-18	Male	48	Tongue	Squamous Cell Carcinoma (SCC)

Supplementary Table 2: List of primer with sequences
Quantitative real-time PCR primers (q-RTPCR)

Serial No.	Gene ID	5’-oligo Sequence-3’
1.	Nup88 F	CTGCTTTGTACTCGAGAAGAT
2.	Nup88 R	GCTGAGGAGCTGAAGGCATTCTTC
3.	Nup62 F	CTACAAGCTGGCTGAGAACA
4.	Nup62 R	ATGTGCGCATTGAGGATCT
5.	Rps16 F	CGCGGCAATGGTCTCATCAAG
6.	Rps16 R	GGAGATGGACTGACGGATAGCA
7.	MKi67 F	AGAGTCAGGTTCAGAAATCC
8.	MKi67 R	TCTTTCTCCCTCCTCTCTT
9.	IL6 F	TACATCCTCGACGGCATCTC
10.	IL6 R	CCAGGCAAGTCTCCTCATTG
11.	c-Myc F	CTGAGGAGGAACAAGAAGATGAG
12.	c-Myc R	TAGTTGTGCTGATGTGTGGAG
13.	Akt F	CAAGGACGGGCACATTAAGA
14.	Akt R	CCGCACATCATCTCGTACAT
15.	BIRC3 F	TTTCCGTGGCTCTTATTCAAACT
16.	BIRC3_R	GCACAGTGGTAGGAACTTCTCAT
17.	Bcl2 F	GGTGGGGTCATGTGTGTGG
18.	Bcl2 R	CGGTTCAGGTACTCAGTCATCC
19.	Actin F	AACTGGGACGACATGGAGAAAA
20.	Actin R	GGATAGCACAGCCTGGATAGCA
